# Spermatic cord metastasis presenting as strangulated inguinal hernia – first manifestation of a multifocal colon adenocarcinoma: a case report

**DOI:** 10.1186/1757-1626-2-61

**Published:** 2009-01-16

**Authors:** Ioannis Galanis, Grigoris Chatzimavroudis, Alexandros Katsougiannopoulos, Nikiforos Galanis, John Makris, Konstantinos Atmatzidis

**Affiliations:** 1 2nd Surgical Department, Aristotle University of Thessaloniki, "G. Gennimatas" General Hospital, Thessaloniki, Greece

## Abstract

Spermatic cord is a rare metastatic site of colorectal cancer. We herein report a case of spermatic cord metastasis of a previous undiagnosed multifocal colon adenocarcinoma, which was clinically presented as a strangulated groin hernia.

## Introduction

Malignant lesions of spermatic cord, both primary and metastatic, are extremely uncommon. The proportion of metastatic neoplasms is very low, as these tumours account for less than 10% of malignant tumors of this site [1]. The most common primary site for spermatic cord metastasis is gastrointestinal tract, followed by pancreas, prostate and kidneys [2,3].

We herein describe the case of a 80-year-old male patient with multifocal colon adenocarcinoma, whose first presentation was a spermatic cord metastasis which was presented as strangulated inguinal hernia.

## Case presentation

**Figure 1:**
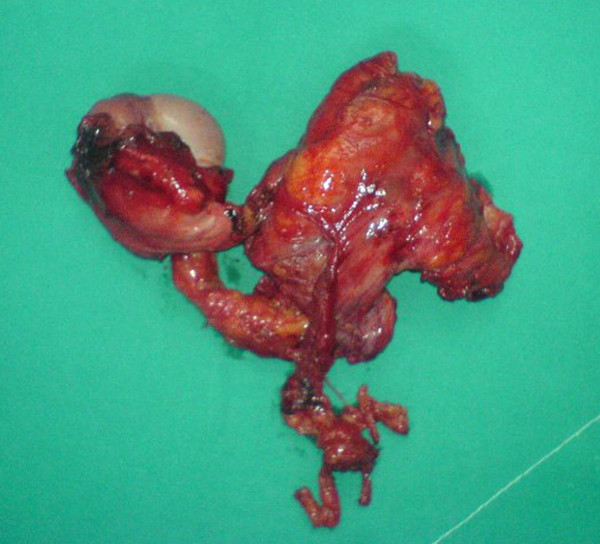
**Surgical specimen showing the spermatic cord tumor**.

An 80-year-old man with a known history of right inguinal hernia was admitted to our department complaining for intermittent pain at the right groin and multiple episodes of vomiting started approximately 48 hours earlier. Clinical examination revealed the presence of a painful mass at the right inguinal area, as well as a tender abdomen with decreased bowel sounds. Abdominal radiography showed the presence of multiple air fluid levels and distended small bowel loops. Based on the medical history of the patient and the clinical examination, the diagnosis of strangulated inguinal hernia was made and the patient was emergently transferred to the operating room for open repair. Through a right inguinal incision the inguinal canal was accessed, where a relatively large, but not incarcerated inguinal hernia was found. In addition a solid mass was observed in close relation to spermatic cord and spermatic vessels. A small piece of the tumor was excised and sent for frozen section examination which was positive for malignancy. The patient underwent right high orchiectomy (Fig. 1), followed by an exploratory laparotomy, as patient's symptoms could not be explained by the findings of the inguinal canal. Investigation of the abdomen revealed the presence of ascites, distended small bowel loops, 3 large tumors located at sigmoid, cecum and ascending colon, as well as multiple smaller lesions across the entire length of the small intestine and peritoneum. Interestingly, cecal mass was in direct contact with the posterior wall of the right inguinal canal. Tissue samples from the tumors of sigmoid and cecum were sent for histopathological examination which identified the presence of multifocal colonic adenocarcinoma. Due to the extension of the disease no other surgical manipulation was performed. The patient died at the early postoperative period due to complications from the primary disease. The histopathological examination of the inguinal mass revealed the presence of a metastatic tumor of the spermatic cord arising from colonic adenocarcinoma.

## Discussion

The first case of spermatic cord tumor was described in the middle of 19^th ^century by Lesauvage [4]. Since then a relative small number of spermatic cord neoplasms have been reported, with most of them being primary in nature. Metastatic lesions of spermatic cord are extremely rare and only case reports have been described. In a retrospective study by Dutt et al which included 13,500 autopsy specimens and 641 biopsy and orchiectomy specimens, only 2 metastatic lesions of spermatic cord, both from a gastric primary, were observed [1]. Most commonly the primary origin of metastatic lesions of spermatic cord is gastrointestinal tract, followed by pancreas, prostate and kidneys [2,3]. Colon is considered to be the main primary site originated from gastrointestinal tract [5], though in Japan the most frequent primary site is stomach [2].

Primary malignant neoplasms spread to spermatic cord mainly via hematogenous or lymphatic route. However, other routes of spread have also been described, including i) retrograde extension through the vas, either along its lumen, or direct extension via the wall of the vas, and ii) transperitoneal seeding through a patent tunica vaginalis [1]. In our case the possibility of hematogenous or lymphatic spread could not be excluded; however, the presence of the cecal tumor in direct contact with the posterior wall of inguinal canal made the local tissue invasion the most likely mode of spread.

To the best of our knowledge, this is the first described case of a patient with spermatic cord metastasis mimicking strangulated hernia. Incarcerated or strangulated inguinal hernia is a common diagnosis for patients presenting with a painful and non-reducible groin mass. However, a plethora of different pathological entities may mimic an incarcerated or strangulated inguinal hernia, including Meckel's diverticulum, inflamed appendix, diverticulitis, ovulating ovary, liposarcoma of spermatic cord, spermatic cord hematoma and pancreatic pseudocyst [6,7]. The consideration of these diseases in the differential diagnosis of a painful groin mass is critical as it may allow more precise diagnosis and more targeted treatment, especially in high risk patients.

## Conclusion

Though rare, metastatic lesion of spermatic cord should be included in the differential diagnosis of a painful groin mass, especially in high risk patients, in order to achieve a more targeted treatment.

## Competing interests

The authors declare that they have no competing interests.

## Authors' contributions

IG was the main surgeon and was major contributor in revising the manuscript critically for important intellectual content. GC, AK and KG analyzed and interpreted the patient data, reviewed the relative literature and were contributors in writing the manuscript.

JM and KA were major contributors in revising the manuscript critically for important intellectual content.

## Consent

Written informed consent was obtained from the patient's relatives for publication of this case report and accompanying images. A copy of the written consent is available for review by the Editor-in-Chief of this journal.
